# *Shigella* Type III Secretion Protein MxiI Is Recognized by Naip2 to Induce Nlrc4 Inflammasome Activation Independently of Pkcδ

**DOI:** 10.1371/journal.ppat.1003926

**Published:** 2014-02-06

**Authors:** Shiho Suzuki, Luigi Franchi, Yuan He, Raul Muñoz-Planillo, Hitomi Mimuro, Toshihiko Suzuki, Chihiro Sasakawa, Gabriel Núñez

**Affiliations:** 1 Department of Pathology and Comprehensive Cancer Center, University of Michigan Medical School, Ann Arbor, Michigan, United States of America; 2 Division of Bacterial Infection Biology, Institute of Medical Science, University of Tokyo, Tokyo, Japan; 3 Lycera Corp., Ann Arbor, Michigan, United States of America; 4 Division of Bacteriology, Department of Infectious Diseases Control, International Research Center for Infectious Diseases, Institute of Medical Science, University of Tokyo, Tokyo, Japan; 5 Department of Molecular Bacteriology and Immunology, Graduate School of Medicine, University of the Ryukyus, Okinawa, Japan; 6 Nippon Institute for Biological Science, Tokyo, Japan; University of Toronto, Canada

## Abstract

Recognition of intracellular pathogenic bacteria by members of the nucleotide-binding domain and leucine-rich repeat containing (NLR) family triggers immune responses against bacterial infection. A major response induced by several Gram-negative bacteria is the activation of caspase-1 via the Nlrc4 inflammasome. Upon activation, caspase-1 regulates the processing of proIL-1β and proIL-18 leading to the release of mature IL-1β and IL-18, and induction of pyroptosis. The activation of the Nlrc4 inflammasome requires the presence of an intact type III or IV secretion system that mediates the translocation of small amounts of flagellin or PrgJ-like rod proteins into the host cytosol to induce Nlrc4 activation. Using the *Salmonella* system, it was shown that Naip2 and Naip5 link flagellin and the rod protein PrgJ, respectively, to Nlrc4. Furthermore, phosphorylation of Nlrc4 at Ser533 by Pkcδ was found to be critical for the activation of the Nlrc4 inflammasome. Here, we show that Naip2 recognizes the *Shigella* T3SS inner rod protein MxiI and induces Nlrc4 inflammasome activation. The expression of MxiI in primary macrophages was sufficient to induce pyroptosis and IL-1β release, which were prevented in macrophages deficient in Nlrc4. In the presence of MxiI or *Shigella* infection, MxiI associated with Naip2, and Naip2 interacted with Nlrc4. siRNA-mediated knockdown of Naip2, but not Naip5, inhibited *Shigella*-induced caspase-1 activation, IL-1β maturation and Asc pyroptosome formation. Notably, the Pkcδ kinase was dispensable for caspase-1 activation and secretion of IL-1β induced by *Shigella* or *Salmonella* infection. These results indicate that activation of caspase-1 by *Shigella* is triggered by the rod protein MxiI that interacts with Naip2 to induce activation of the Nlrc4 inflammasome independently of the Pkcδ kinase.

## Author Summary

*Shigella* are bacterial pathogens that are the cause of bacillary dysentery. An important feature of *Shigella* is their ability to invade the cytoplasm of host epithelial cells and macrophages. A major component of host recognition of *Shigella* invasion is the activation of the inflammasome, a molecular platform that drives the activation of caspase-1 in macrophages. Although *Shigella* is known to induce the activation of the Nlrc4 inflammasome, the mechanism by which the bacterium activates Nlrc4 is largely unknown. We discovered that the *Shigella* T3SS inner rod protein MxiI induces Nlrc4 inflammasome activation through the interaction with host Naip2, which promoted the association of Naip2 with Nlrc4 in macrophages. Expression of MxiI induced caspase-1 activation, Asc oligomerization, pyroptosis and IL-1β release which required Naip2, but not Naip5. Significantly, caspase-1 activation induced by *Shigella* infection was unaffected by deficiency of the Pkcδ kinase. This study elucidates the microbial-host interactions that drive the activation of the Nlrc4 inflammasome in *Shigella*-infected macrophages.

## Introduction

Recognition of intracellular pathogenic bacteria by members of the nucleotide-binding domain and leucine-rich repeat containing (NLR) family triggers immune responses against bacterial infection [1], [2]. A major response against several pathogenic Gram-negative bacteria, including *Salmonella*, *Legionella*, and *Shigella* is the activation of caspase-1 via Nlrc4 in macrophages [1], [3]. Upon bacterial stimulation, Nlrc4 mediates the formation of a multi-protein complex termed the inflammasome that induces the activation of caspase-1 leading to the proteolytic maturation of pro-IL-1β and pro-IL-18 as well as the induction of pyroptotic cell death in macrophages [4]–[6]. Many Gram-negative bacteria encode a type III secretion system (T3SS) with conserved structural features that promote virulence by injecting bacterial effector proteins directly into the cytosol of host cells [7], [8]. In macrophages infected with *Salmonella*, the cytosolic delivery of flagellin or the bacterial rod protein PrgJ through the T3SS is recognized by Nlrc4 leading to inflammasome activation [9]. Recently, Naips (NLR family, apoptosis inhibitory proteins) have been shown to act as adaptor molecules that connect flagellin or the bacterial rod protein PrgJ to Nlrc4 [10], [11]. Specifically, Naip5 and Naip6 associate with flagellin to promote Nlrc4 oligomerization and inflammasome activation, whereas Naip2 links PrgJ to Nlrc4 [10]–[12]. These findings suggest a model in which certain Naips specifically recognize flagellin or PrgJ to mediate Nlrc4 inflammasome activation. Recent studies, however, have revealed that the activation of Nlrc4 is more complex in that phosphorylation of Nlrc4 at Ser533 was found to be critical for the activation of the inflammasome [13]. Furthermore, it was suggested that Pkcδ is the major Nlrc4 kinase responsible for Nlrc4 phosphorylation and inflammasome activation [13].

*Shigella* are non-flagellated bacterial pathogens that contain highly evolved invasion systems that enable them to invade host cells and colonize the epithelium of the large intestine, which ultimately leads to a severe form of colitis called bacillary dysentery [14]. After uptake of *Shigella* by intestinal macrophages, the bacterium delivers a subset of effector proteins via the T3SS apparatus into the host cytosol [7], [8], [15]. The inner rod of the T3SS needle complex forms a conduit for protein transport through the periplasm which is assembled by the polymerization of PrgJ in *Salmonella* and its homologue MxiI in *Shigella* [16], [17]. Because of the homology of *Salmonella* PrgJ with *Shigella* MxiI, it can be predicted that *Shigella* induces activation of Nlrc4 via the sensing of MxiI by host macrophages. Consistent with this notion, the T3SS of *Shigella* is required to induce IL-1β secretion and pyroptosis via the Nlrc4 inflammasome [18]. Furthermore, ectopic expression of MxiI reduced the viability of macrophages and this was inhibited in the absence of Nlrc4 [9]. However, the mechanism by which *Shigella* MxiI induces activation of the Nlrc4 inflammasome remains unknown. In this study, we provide evidence that MxiI mediates the activation of the Nlrc4 inflammasome through interactions with Naip2. Furthermore, we demonstrate that Naip2, but not Naip5, is critical for the interaction of MxiI with Nlrc4 and the activation of the inflammasome in macrophages infected with *Shigella*. Finally, we show that Pkcδ is dispensable for Nlrc4 activation.

## Results

### Expression of *Shigella* rod protein MxiI induces activation of the Nlrc4 inflammasome

**Figure 1:**
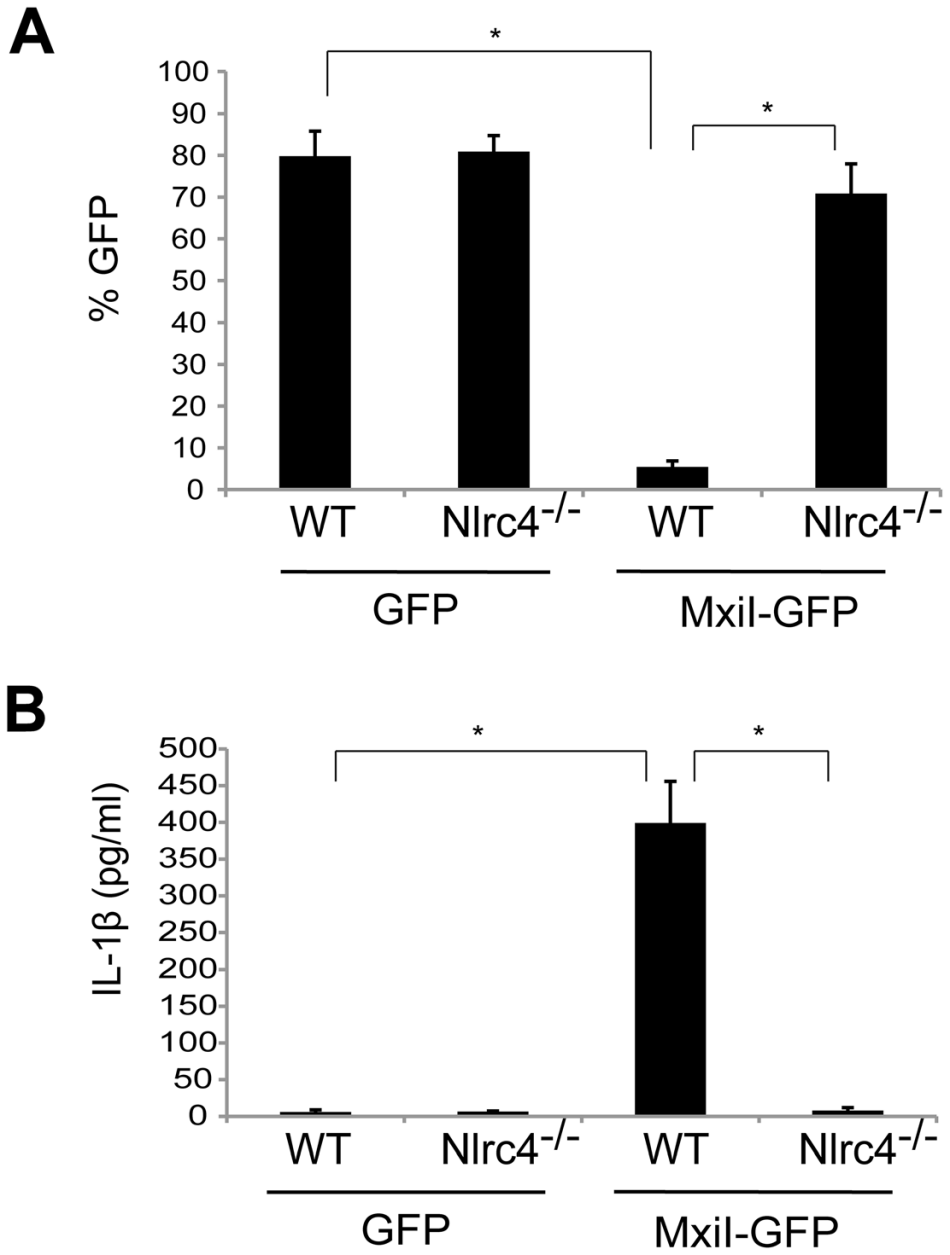
Expression of *Shigella* rod protein MxiI induces activation of the Nlrc4 inflammasome in macrophages. WT or *Nlrc4* ^−/−^ BMDMs were nucleofected with MSCV-IRES-GFP (GFP) or MSCV-IRES-GFP encoding *Shigella* MxiI (MxiI-GFP). After 20 hrs, the percentage of GFP-positive viable cells in the total cell population was analyzed by fluorescence microscopy (**A**) and the production of IL-1β in cell free supernatants by ELISA (**B**). * p<0.0001. (**A** and **B**) Results represent mean ± SD and are representative of three independent experiments.

In the case of flagellated pathogenic bacteria, flagellin is a major and potent stimulator of the Nlrc4 inflammasome. In addition, *Salmonella* T3SS rod protein PrgJ is sensed by Nlrc4 to activate caspase-1. Because *Shigella* are unflagellated bacteria, we hypothesized that the *Shigella* T3SS rod protein MxiI, a homologue of *Salmonella* PrgJ, induces the activation of the Nlrc4 inflammasome. To test this hypothesis, we expressed MxiI in wild-type (WT) and Nlrc4-deficient bone marrow-derived macrophages (BMDM) using a MSCV-IRES-GFP retroviral vector and assessed cell viability by the numbers of viable green fluorescence protein (GFP)-positive cells. After overnight culture, the viability of WT macrophages was dramatically decreased by MxiI-GFP expression when compared to expression of GFP (Figure 1A). Importantly, the decrease in cell viability was inhibited in Nlrc4^−/−^ macrophages (Figure 1A). Consistently, expression of MxiI-GFP, but not GFP, induced the release of IL-1β in WT macrophages, which was abolished in macrophages lacking Nlrc4 (Figure 1B). These results indicate that expression of MxiI induces the activation of the Nlrc4 inflammasome.

### *Shigella* MxiI interacts with Naip2 and promotes the interaction of Naip2 with Nlrc4

**Figure 2:**
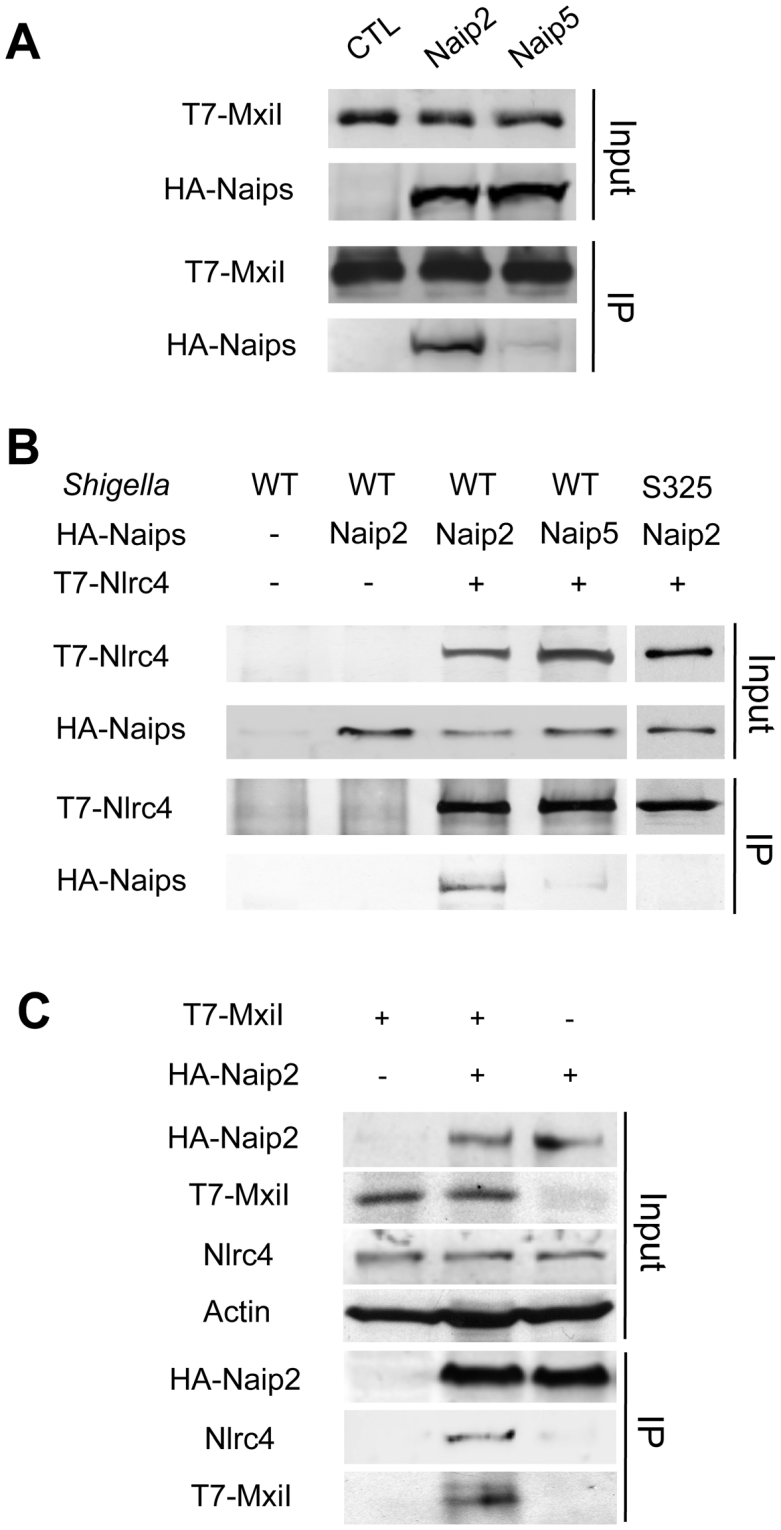
*Shigella* MxiI interacts with Naip2 and promotes the interaction of Naip2 with Nlrc4. (A)T7-tagged MxiI was co-expressed with HA-tagged Naip2 or Naip5 or control empty vector in caspase-1-deficient BMDMs. Cell lysates were immunoprecipitated with anti-T7 antibody and the interaction between MxiI and Naip2/5 was analyzed by immunoblotting with anti-HA antibody. (B) T7-tagged Nlrc4 or empty vector was co-expressed with HA-tagged Naip2, Naip5, or empty vector in caspase-1-deficient BMDMs. After 16 hrs cells were infected with *Shigella* WT or S325 mutant for 2 hr at a bacteria/macrophage ratio of 10∶1. Cell lysates were immunoprecipitated with anti-T7 beads and the interaction between Nlrc4 and Naip2/5 was analyzed by immunoblotting with anti-HA antibody. (C) T7-tagged MxiI or empty vector was co-expressed with HA-tagged Naip2. Cell lysates were immunoprecipitated with anti-HA beads and the interaction of Naip2 with MxiI and endogenous Nlrc4 was analyzed by immunoblotting with anti-T7 or anti-Nlrc4 antibody. (A–C) Results are representative of three independent experiments.

We next tested whether the rod protein MxiI interacts with Naip2 or Naip5 in macrophages. Because expression of MxiI in macrophages causes cell death (Figure 1A), we used macrophages from caspase-1-deficient mice to assess the interaction of MxiI with Naip proteins by immunoprecipitation. In these experiments, we expressed T7-tagged MxiI in the presence of HA-tagged Naip2, HA-tagged Naip5 or control plasmid. Immunoprecipitation analysis showed that MxiI associated with Naip2, but much less with Naip5 as revealed by immunoblotting with anti-HA antibody (Figure 2A). Next, we investigated the interaction between Nlrc4 and Naip2 in *Shigella*-infected macrophages. To assess this, we expressed T7-tagged Nlrc4 and HA-tagged Naip2 or Naip5, or control empty vector in uninfected or caspase-1-deficient macrophages infected with WT or an isogenic *Shigella* strain deficient in the T3SS (S325). Immunoprecipitation analysis revealed that Naip2 interacts with Nlrc4 in macrophages infected with WT *Shigella* (Figure 2B). However, Naip2 did not associate with Nlrc4 in uninfected macrophages or macrophages infected with the mutant bacterium lacking a functional T3SS that are unable to release MxiI into the host cytosol (Figure 2B). Furthermore, infection with *Shigella* preferentially promoted the interaction of Nlrc4 with Naip2 relative to Naip5 (Figure 2B). MxiI is secreted into the culture medium by *Shigella* which relies on the presence of a functional T3SS [19]–[21]. Therefore, MxiI is presumably leaked into the host cytosol via the T3SS to activate Nlrc4, as it was suggested for *Salmonella* PrgJ [22], [23]. Therefore, we next asked whether expression of MxiI promotes the association of Naip2 with endogenous Nlrc4 in uninfected macrophages. Immunoprecipitation experiments showed that expression of MxiI induced the interaction of Naip2 with endogenous Nlrc4 (Figure 2C). Collectively, these results indicate that MxiI interacts preferentially with Naip2 and promotes the interaction between Naip2 and Nlrc4.

### Naip2 promotes the processing of IL-1β in an inflammasome reconstitution system

We next performed additional studies to verify that *Shigella* infection promoted the activation of Nlrc4 via Naip2. To confirm the preferential effect of Naip2 on Nlrc4 activation, we performed reconstitution experiments by expressing Nlrc4, Asc, caspase-1, pro-IL-1β and Naip2 or Naip5 in 293T cells. One day after transfection, cells were infected with WT or T3SS-deficient *Shigella* for 3 hrs and inflammasome activation was analyzed by immunoblotting with an antibody specific for mature IL-1β p17. In the absence of exogenous Naip2 or Naip5, infection with WT *Shigella* enhanced the processing of pro-IL-1β into IL-1β p17 (Figure S1A). The formation of IL-1β p17 was further enhanced by Naip2, but inhibited by Naip5 in *Shigella*-infected cells (Figure S1A). In this reconstitution system, the enhancement of IL-1β p17 formation by Naip2 in cells infected with WT *Shigella* required Nlrc4, Asc and caspase-1 (Figure S1B).

### *Shigella* induces caspase-1 activation via Nlrc4, Asc and Naip2 in macrophages

*Shigella* infection stimulates Nlrc4- and Asc-dependent inflammasome activation in macrophages [18]. However, *Shigella* was also shown to induce macrophage cell death via Nlrp3 after 2–6 hrs of infection at a bacteria/macrophage ratio of 50∶1 [24]. To verify these seemingly contradictory results, we reassessed the role of Asc, Nlrc4 and Nlrp3 in *Shigella*-induced caspase-1 activation. In these experiments, LPS-primed BMDM were infected with the *Shigella* WT or S325 (T3SS-deficient mutant) at a bacteria/macrophage ratio of 10∶1 for 30 min. As expected, WT, but not mutant *Shigella*, induced processing of procaspase-1 into the p20 subunit of caspase-1 (Figure S2A). The inability of the mutant bacterium to activate caspase-1 could not be explained by reduced uptake by macrophages (Figure S3). Importantly, caspase-1 activation, IL-1β release, and pyroptosis required Nlrc4 and Asc, but not Nlrp3 (Figure S2A–C). Because previous studies showed that Asc was not required for pyroptosis induced by *Shigella* in BMDM differentiated for 5 days [18], we assessed cell death induced by *Shigella* in BMDM differentiated for 3, 4 and 5 days in culture (Figure S4). Consistent with previous studies [18], Asc was not required for pyroptosis in macrophages differentiated for 5 days (Figure S4). In macrophages differentiated for 3 or 4 days, however, cell death induced by *Shigella* was enhanced in WT macrophages and impaired in Asc-deficient macrophages (Figure S2C and S4) which is in line with the results presented in Figure S2C.

**Figure 3:**
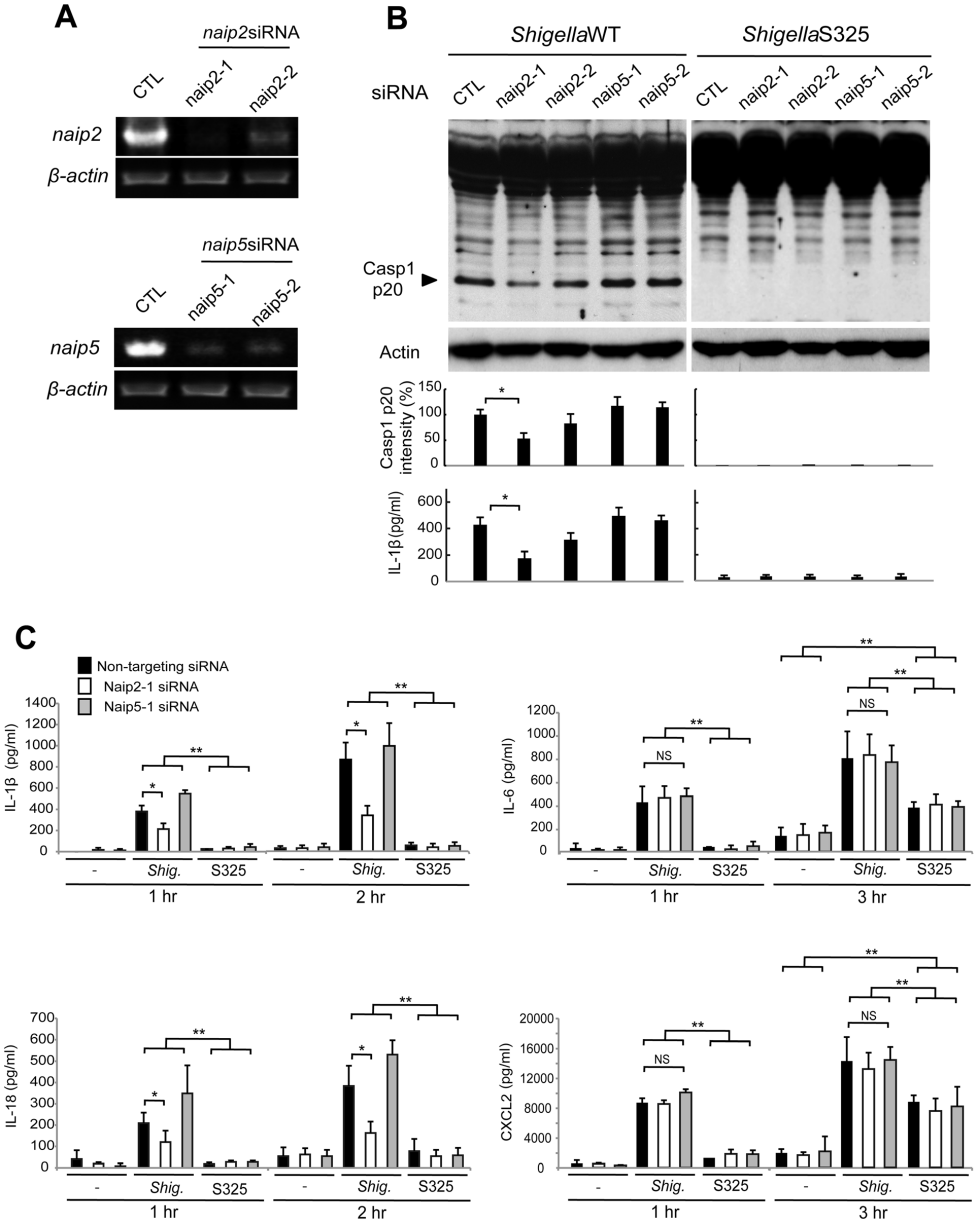
*Shigella* induces caspase-1 activation via Nlrc4, Asc and Naip2 in macrophages. (A–C). BMDM were nucleofected with siRNA targeting Naip2 or Naip5 for 48 hrs and then infected, or not, with *Shigella* WT or S325 mutant for additional 1–3 hrs at a bacteria/cell ratio of 10∶1. (**A**) Knockdown efficiency was evaluated by RT-PCR after 48 hrs in uninfected cells. (**B** and **C**) The activation of caspase-1 was evaluated by detecting cleaved caspase-1 (p20) by immunoblotting, and the level of casp1 p20 was further quantified by densitometry (**B**). Cytokines production was analyzed by ELISA in cell free supernatants (**B–C**). * p<0.05. **p<0.001; NS, not significant. (**A**–**C**) Results represent mean ± SD and are representative of three independent experiments.

Next, we investigated the role of Naip2 and Naip5 in caspase-1 activation induced by *Shigella*. We used siRNA-mediated knockdown to reduce the expression of Naip2 and Naip5 in macrophages (Figure 3A). Notably, caspase-1 activation induced by *Shigella* was attenuated by inhibiting the expression of Naip2, but not Naip5 (Figure 3B). Importantly, the ability of individual siRNA to inhibit caspase-1 activation correlated with reduction of Naip2 expression (Figure 3A, B). In addition, knockdown of Naip2, but not Naip5, reduced the release of IL-1β and IL-18 induced by *Shigella* infection at 1 or 2 hrs post-infection (Figure 3B, C). In control experiments, knockdown of Naip2 did not affect the production of IL-6 or CXCL2 in macrophages infected with WT or S325 mutant *Shigella* (Figure 3C). These results suggest that *Shigella* induces Nlrc4-dependent inflammasome activation via Naip2 in macrophages.

### *Shigella* or MxiI expression induces Naip2-dependent Asc pyroptosome formation in macrophages

**Figure 4:**
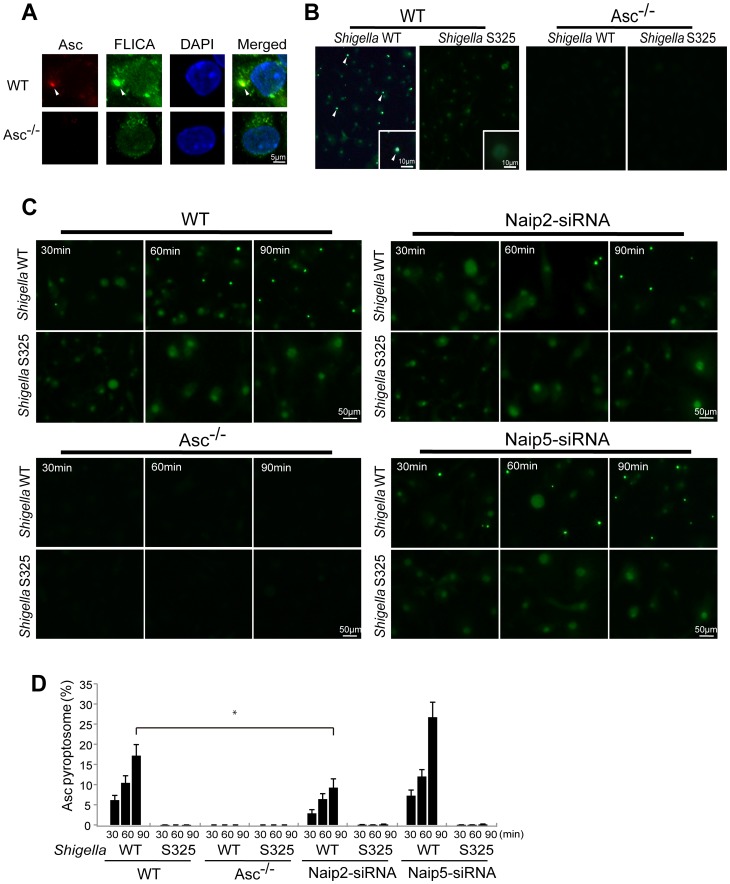
*Shigella* induces Naip2-dependent Asc pyroptosome formation in macrophages. WT (**A–D**), *Asc* ^−/−^ (**A–D**), Naip2-deficient (siRNA) or Naip5-deficient (siRNA) (**C**) BMDM were infected with *Shigella* WT or S325 mutant for up to 90 min (**A**–**D**). Cells were fixed and analyzed by confocal microscopy (**A**–**D**) and the percentage of cells containing Asc pyrotopsomes was evaluated (**D**). Caspase-1 activation was detected using FLICA reagent (**A**) (green), Asc localization with anti-Asc antibody (red in **A**, green in **B** and **C**) and nuclei with DAPI (**A**) (blue). Arrows denotes Asc pyroptosomes. * p<0.01. Results represent mean ± SD and are representative of three independent experiments.

**Figure 5:**
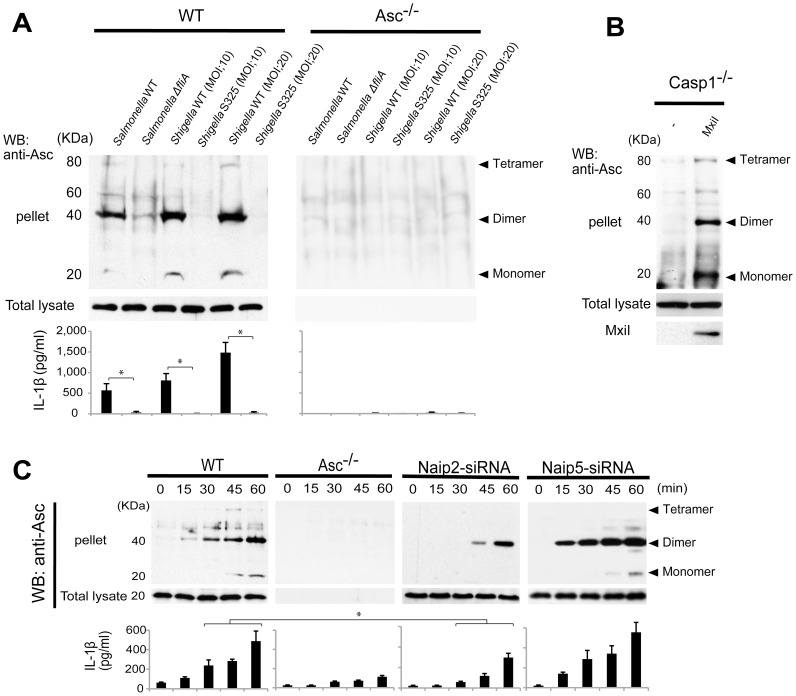
*Shigella* or MxiI expression induces Naip2-dependent Asc dimerization in macrophages. WT (**A** and **C**), *Asc* ^−/−^ (**A** and **C**), *casp1* ^−/−^ (**B**), Naip2-deficient (siRNA) or Naip5 deficient (siRNA) (**C**) BMDM were infected with *Shigella* WT or S325 *Shigella* mutant, *Salmonella* WT or *ΔfliA Salmonella* mutant at the indicated bacteria/cell ratio for up to 60 min (**A** and **C**), or nucleofected with pCMV producing, or not, MxiI (**B**). The dimerization of Asc in the insoluble fraction (pellet) was evaluated by immunoblotting with anti-Asc antibody (**A**–**C**) and the production of IL-1β in cell free supernatants was analyzed by ELISA (**A** and **C**). Middle panel shows the presence of Asc in total cell lysate. Results represent mean ± SD. Representative of three independent experiments. *p<0.002 (**A**), *p<0.05 (**C**).

The Asc pyroptosome is a molecular platform that is thought to be important for the recruitment and activation of caspase-1 [25]–[27]. Infection of macrophages with WT, but not T3SS-deficient, *Shigella* induced the formation of the Asc pyroptosome which was detected in the cell cytoplasm by staining with an antibody that recognizes Asc (Figure 4A, B). The Asc pyroptosome induced by *Shigella* infection co-localized with FLICA staining that labels activated caspase-1 (Figure 4A). Importantly, knockdown of Naip2 by siRNA reduced Asc pyroptosome formation whereas Naip5 did not (Figure 4C, D). To provide direct biochemical evidence that the Asc pyroptosome is formed, we cross-linked the insoluble Asc protein complexes from *Shigella* or *Salmonella* infected macrophages and subjected them to immunoblotting with anti-Asc antibody. Immunoblotting analysis revealed that infection with WT *Shigella* or *Salmonella* induces prominent Asc dimer formation in WT, but not Asc-deficient macrophages (Figure 5A, upper panel). The induction of Asc dimers correlated with IL-1β release in culture supernatants (Figure 5A, lower panel). In contrast, *Shigella* deficient in T3SS and the *fliA*-deficient *Salmonella* mutant were impaired in the induction of Asc dimer formation (Figure 5A). Notably, expression of MxiI was sufficient to induce the formation of Asc dimers in caspase-1-deficient macrophages in the absence of *Shigella* infection (Figure 5B). Furthermore, knockdown of Naip2 by siRNA, but not Naip5, inhibited Asc dimer formation (Figure 5C). These results indicate that *Shigella* MxiI and Naip2 are important in Asc pyroptosome formation which is associated with inflammasome activation.

### *Shigella* induces IL-1β secretion independently of Pkcδ

**Figure 6:**
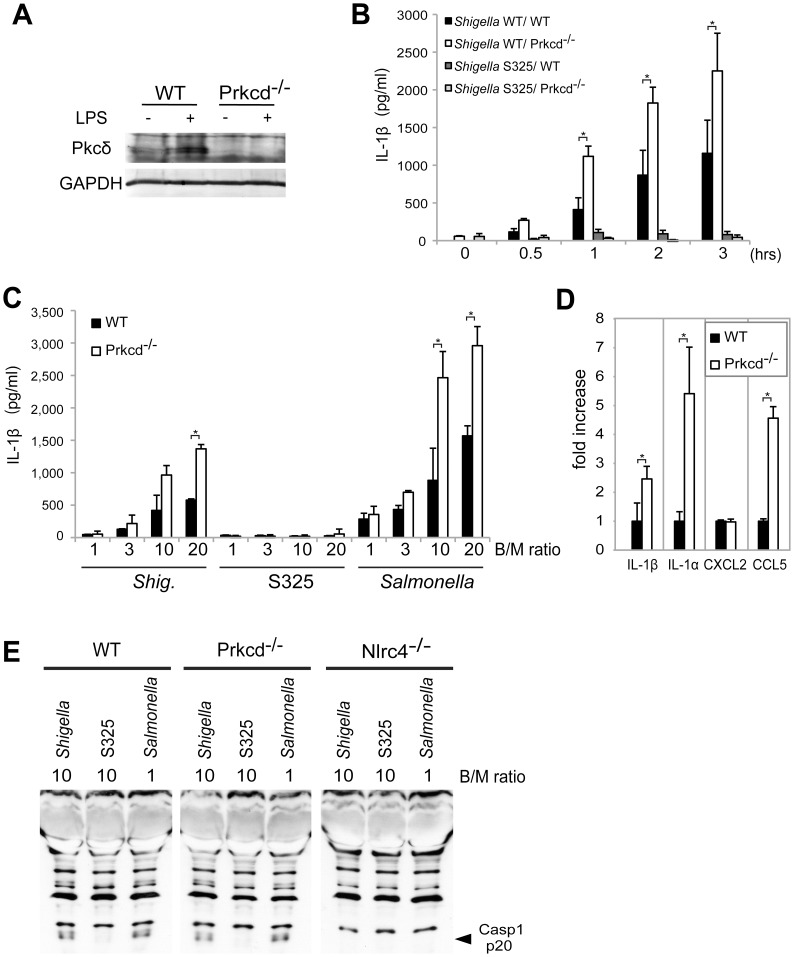
Pkcδ is not required for inflammasome activation caused by *Shigella* infection. (**A**) BMDMs from WT and *Prkcd* ^−/−^ mice were stimulated with LPS and the expression of Pkcδ was evaluated by immunoblotting. (**B–D**) BMDMs from WT and *Prkcd* ^−/−^ mice were infected with *Shigella* WT or S325 *Shigella* mutant (**B–E**) or *Salmonella* (**C and E**) at a bacteria/macrophage ratio of 10∶1 for various time points (**B**) or at the indicated bacteria/macrophage ratio (**C**) for 1 hr, or with indicated bacteria/macrophage ratios for 2 hrs (**D**) or 30 min (**E**). The production of cytokines in cell free supernatant was analyzed by ELISA (**B–D**) and the activation of caspase-1 was evaluated by detecting cleaved caspase-1 (p20) by immunoblotting (**E**). *p<0.02. Results represent mean ± SD. Results are representative of at least three independent experiments.

Recent studies reported that Nlrc4 phosphorylation by Pkcδ is critical for inflammasome activation induced by *Salmonella* infection [13]. Thus, we assessed whether inflammasome activation caused by *Shigella* infection also requires Pkcδ. In these experiments, LPS-primed BMDM from WT and Pkcδ-deficient mice were infected with WT or S325 (T3SS-deficient mutant) *Shigella*, and IL-1β release was evaluated at different time points and bacterial/macrophage ratios after infection. As expected, expression of Pkcδ was induced by LPS stimulation in WT, but not Pkcδ-deficient macrophages (Figure 6A). Importantly, Pkcδ was not required for IL-1β secretion induced by *Shigella* or *Salmonella* (Figure 6B–D). In fact, Pkcδ deficiency enhanced IL-1β secretion in response to *Shigella* and *Salmonella* infection (Figure 6B–D). Furthermore, Pkcδ-deficient macrophages produced higher amounts of IL-1α and CCL5, but not CXCL2 than WT macrophages in response to infection (Figure 6D). The increased production of cytokines in Pkcδ-deficient macrophages was not associated with enhanced NF-κB or MAPK activation after *Shigella* infection (Figure S5). Notably, induction of apoptosis in *Shigella*-infected macrophages was inhibited in macrophages deficient in Pkcδ (Figure S5). Furthermore, treatment with z-DEVD-fmk, a cell permeable caspase-3 inhibitor, increased the production of IL-1β in WT macrophages infected with *Shigella* (Figure S5), suggesting that increased production of IL-1β in Pkcδ-deficient macrophages is mediated, at least in part, by inhibition of apoptosis in *Shigella*-infected macrophages. Importantly, caspase-1 activation induced by *Shigella* or *Salmonella* was unimpaired in macrophages deficient in Pkcδ (Figure 6E), whereas it was abolished in macrophages deficient in Nlrc4 (Figure 6E). These results indicate that Pkcδ is not essential for inflammasome activation induced by *Shigella* or *Salmonella* infection.

## Discussion

The intracellular sensing of flagellin is the major trigger for the activation of the Nlrc4 inflammasome in macrophages infected with *Salmonella* [4]. Because *Shigella* is non-flagellated, the current studies were aimed at understanding the mechanism by which *Shigella* induces the activation of Nlrc4 in macrophages. We show here that *Shigella* induces the activation of the Nlrc4 inflammasome through MxiI, an inner rod protein of the T3SS. MxiI associated with Naip2 and was sufficient to induce Nlrc4-dependent IL-1β secretion and the interaction with Nlrc4. Importantly, inhibition of Naip2 expression impaired the activation of the Nlrc4 inflammasome and IL-1β/IL-18 release in *Shigella*-infected macrophages. Because IL-1β secretion induced by *Shigella* was not abolished by Naip2 knockdown, it is possible that *Shigella* also activates another inflammasome pathway that is minor and only unmasked by the inhibition of the Naip2-Nlrc4 pathway. Alternatively, it is possible that the partial inhibition of IL-1β secretion reflects residual Naip2 protein expression in macrophages.

Our work is consistent with a model in which the T3SS inner rod proteins including PrgJ in *Salmonella* and MxiI in *Shigella* are recognized by Naip2 and this interaction leads to the recruitment and activation of Nlrc4. Consistent with this model, we show that expression of MxiI promotes the association of Naip2 with Nlrc4 and induces the oligomerization of Asc in macrophages. Furthermore, WT, but not T3SS-deficient *Shigella*, enhances the association of Naip2 and Nlrc4 in macrophages. The failure of mutant *Shigella* to induce the interaction between Naip2 and Nlrc4 is presumably explained by the inability of the T3SS mutant to release MxiI into the host cytosol. A measure of inflammasome activation is the formation of Asc oligomers [25]–[27]. Importantly, Asc oligomerization induced by MxiI was observed in caspase-1-deficient macrophages, indicating that this critical event is not a secondary event of caspase-1 activation. MxiI is composed of 97 amino acids and is predicted to be a soluble protein using publically available tools (http://www.psort.org/psortb). It has been shown that MxiI is secreted into the culture medium by *Shigella* in a T3SS dependent manner [19]–[21]. Thus, as it was suggested for *Salmonella* PrgJ [9], [22], we propose that small amounts of MxiI are leaked into the host cytosol via the T3SS during *Shigella* infection to induce the activation of Nlcr4.

Recent studies showed that Nlrc4 phosphorylation was induced by *Salmonella* and was found to be critical for inflammasome activation [13]. Furthermore, it was proposed that Pkcδ was the major kinase responsible for phosphorylation of Nlrc4 [13]. In contrast to the latter finding, we found that IL-β secretion and caspase-1 activation induced by *Shigella* and *Salmonella* infection were not impaired in Pkcδ-deficient macrophages. Notably, the production of several cytokines including IL-1β was enhanced in infected Pkcδ-deficient macrophages. A possible mechanism to account for the enhanced production of cytokines in Pkcδ-deficient macrophages is the observation that Pkcδ regulates phagosomal production of ROS [28] which is known to inhibit pro-inflammatory responses including cytokine production [29]. However, we did not observe enhanced NF-κB or MAPK activation in Pkcδ-deficient macrophages infected with *Shigella*. Pkcδ has been shown to regulate the induction of apoptosis [30]–[32]. Consistently, apoptosis induced by Shigella infection was impaired in Pkcδ-deficient macrophages and treatment with a caspase-3 inhibitor enhanced IL-1β secretion in WT macrophages. These results suggest that the increased production of cytokines observed in Pkcδ-deficient macrophages might be due, at least in part, to suppression of apoptosis in infected macrophages. Regardless of the mechanism involved, our results clearly show that caspase-1 activation induced by *Shigella* or *Salmonella* infection is not impaired in Pkcδ-deficient macrophages. We do not have a clear explanation for the difference in results between our studies and previous results by Qu et al. These authors showed that in addition to Pkcδ, Pak2 was capable of phosphorylating Nlrc4 at the critical Ser533, although the results suggested that Pak2 was a minor Nlrc4-phosphorylating kinase [13]. Thus, it is conceivable that the difference in results could be explained by kinase redundancy and subtle variation in the expression of Nlrc4-phosphorylating kinases in different macrophage preparations. Regardless of the explanation, findings within this investigation clearly show that Pkcδ is dispensable for Nlrc4 activation. Thus, our results challenge the notion that Pkcδ is critical for inflammasome activation and indicate that further work is needed to understand the mechanism and role of Nlrc4 phosphorylation in inflammasome activation.

*Shigella* MxiI associates with Naip2 to induce the interaction of Naip2 with Nlrc4, which presumably leads to Nlrc4 oligomerization and inflammasome activation. In the *Salmonella* system, cytosolic flagellin binds to Naip5 and induces the association of Naip5 with Nlrc4 [10]–[12]. Reconstitution experiments with purified flagellin, Naip5 and Nlrc4 revealed that these components are sufficient to induce the formation of a disk-like complex composed of 11 or 12 proteins including Nlrc4 and Naip5, although the exact ratio of Naip5 and Nlrc4 in the complex remains unclear [12]. Based on the latter observations, we suggest that *Shigella* MxiI induces the oligomerization of Nlrc4 via their interaction with Naip2. Consistent with this model, we found that MxiI induced the interaction of Naip2 with Nlrc4 and the oligomerization of Asc. Furthermore, Naip2, but not Naip5, was critical for caspase-1 activation, pyroptosome formation, Asc oligomerization and IL-1β secretion. Collectively, these results support a model in which distinct Naip family members act as sensors of flagellin and T3SS inner rod proteins and oligomerized Nlrc4 provides a platform for the recruitment and activation of caspase-1. While Naip2 knockdown reduced inflammasome activation, Naip5 knockdown had the opposite effect in response to *Shigella* infection. Although further work is needed to understand the role of Naip5, one possibility is that there is competition between Naip2 and Naip5 protein complexes and inhibition of Naip5 enhances the Naip2-Nlrc4 inflammasome pathway. Nlrc4 and caspase-1 contain CARD domains and they could interact directly via homotypic CARD-CARD interactions. However, the adaptor Asc is essential for the activation of caspase-1 in response to *Salmonella* and *Shigella* [18], [33]. These results suggest that Asc is somehow required for the interaction between Nlrc4 and caspase-1 or that Asc is critical for another step which is important for inflammasome activation.

## Materials and Methods

### Ethic statement

All animal experiments were conducted according to the U.S.A. Public Health Service Policy on Humane Care and Use of Laboratory Animals. Animals were maintained in an AAALAC approved facility and all animal studies followed protocol 09716-2 that was approved by the Animal Care and Use Committee of the University of Michigan (Ann Arbor, MI).

### Mice

Mice deficient in Nlrc4, Nlrp3, Asc and caspase-1/11 have been previously described [4], [34], [35]. All mice were crossed at least 5 times on a C57BL/6 background. Bone marrow samples from *Prkcd* ^−/−^ mice in C57BL/6 background were provided by Hee-Jeong Im Sampen (Rush University Medical Center, Chicago, IL).

### Bacterial strains and plasmids

*Shigella flexneri* strain YSH6000 [36] was used as the WT strain, and S325 (mxiA::Tn5) [37] was used as the T3SS–deficient control. The WT *S. enterica* serovar Typhimurium SR-11 χ3181 and the isogenic fliA::Tn10 were provided by H. Matsui (Kitasato Institute for Life Science, Tokyo, Japan) [38]. *ΔfliA Salmonella* mutant is impaired in the expression of flagellin [18]. cDNAs encoding mouse Naip2, Naip5, Nlrc4, Asc, caspase-1, and bacterial MxiI were amplified by PCR and cloned into the pCMV based mammalian expression vector or the MSCV-IRES-GFP retroviral expression vector (Addgene). Human pro-IL-1β clone (RDB6666) was provided by RIKEN BRC which is participating in the National Bio-Resource Project of the MEXT, Japan.

### Cell culture and reagents, antibodies

BMDMs were prepared from the femurs and tibias of mice and cultured for 3–7 days in 10% FCS IMDM (Gibco) supplemented with 30% L-cell supernatant, non-essential amino acids, sodium pyruvate and antibiotics (Penicillin/Streptomycin). 293T cells were cultured on Dulbecco's Modified Eagle's medium (Sigma) containing 10% FCS and antibiotics (Penicillin/Streptomycin). The rabbit anti mouse caspase-1 p20 and anti-mouse Nlrc4 antibodies were produced in our laboratory by immunizing rabbits with mouse caspase-1 (p20 subunit) and mouse Nlrc4 (amino acids 1–152) recombinant proteins [39]. Anti–IL-1β p17 (#2021) and anti-Pkcδ (#2058) antibodies were from Cell Signaling. Mouse monoclonal anti-β-actin antibody was from Sigma. HRP-conjugated goat anti–rabbit (Jackson Laboratories) or anti–mouse IgG (Sigma) or anti-rat (Jackson Laboratories), or AP-conjugated goat anti-rabbit (Santa Cruz Biotechnology Inc.) or anti-mouse IgG (Santa Cruz Biotechnology Inc.) antibodies were used as secondary antibodies for immunoblotting.

### Bacterial infection

Macrophages were seeded in 24-well plates at a density of 3×10^5^ cells per well. Cells were stimulated with or without 0.1 µg/ml LPS (from *E. coli* O55:B5, Sigma) for 6 h and then infected with *Shigella* or *Salmonella*. Bacterial strains were pre-cultured overnight in Mueller-Hinton broth (Difco) at 30°C, then were inoculated into brain heart infusion broth (Difco) and incubated for 2 h at 37°C prior to infection. The cells were infected with *Shigella* at a bacteria/macrophage ratio of 10∶1, or with *Salmonella* at a bacteria/macrophage ratio of 1∶1 unless otherwise stated. The plates were centrifuged at 700 *g* for 5 min to synchronize the infection, and gentamicin (100 µg/ml) and kanamycin (60 µg/ml) were added after 20 min. At the indicated times after infection, cytokines were measured in culture supernatants by enzyme-linked immunoabsorbent assay (ELISA) kits (R and D Systems). RNA was isolated with E.Z.N.A. TM total RNA kit (Omega Biotek) according to the manufacturer's instructions. RNA was reverse transcribed using the High Capacity RNA-to cDNA kit (Applied Biosystem) and cDNA was then used for RT-PCR. For immunofluorescence studies, the infected cells were fixed and immunostained, and then analyzed with a confocal laser-scanning microscope (LSM510; Carl Zeiss) or fluorescence microscopy (Olympus). Carboxyfluorescein FLICA (Immunochemistry Technologies, LLC) was added 1 hr before bacterial infection. Apoptosis was measured by the AnnexinV (Roche) and TUNEL (Promega) assays using fluorometric protocols according to the manufacture's recommendations. For the caspase-3 inhibitor studies, the cells were treated with 200 µM z-DEVD-fmk (Calbiochem) for 1 h before bacterial infection.

### Reconstitution of the NLRC4 inflammasome in 293T cells

293T cells were seeded in 6-well plates at a density of 5×10^5^ cells per well and incubated overnight. Then, the cells were transfected with or without 1 µg T7-tagged Nlrc4, 1 µg T7-tagged Asc, 0.4 µg HA-tagged caspase-1, and 0.4 µg FLAG-tagged proIL-1β [40], and 1 µg HA-tagged Naip2 or Naip5, using FuGENE 6 (Roche). Cells were infected one day after infection. Intensities of casp1 p20 or IL-1β p17 bands were quantified by densitometry, the values normalized to the β-actin protein levels and results were analyzed with ImageJ software.

### Cell survival assays

*The Shigella MxiI* gene was cloned into the MSCV-IRES-GFP retrovirus vector, which contains an IRES-GFP element to track retroviral infection. WT or Nlrc4^−/−^ BMDMs were immortalized using the J2 virus to increase nucleofection efficiency [41]. Then, cells were nucleofected with MSCV-IRES-GFP or MSCV-IRES-GFP encoding *Shigella* MxiI using an Amaxa nucleofector system (Nucleofector kit V and the D-032 program). After 20 hrs, cell survival in the GFP-positive cell population was analyzed by fluorescence microscopy. The LDH activity in the culture supernatants of infected cells was measured using the CytoTox 96 assay kit (Promega) according to the manufacturer's protocol. Assays were performed in triplicate for each independent experiment.

### Bacterial invasion assay

The invasion efficiency of *Shigella* strains was evaluated using a gentamicin/kanamycin protection assay. Briefly, cells were infected for 20 min and then incubated for 20 min at 37°C in medium containing gentamicin (100 µg/ml) and kanamycin (60 µg/ml) to kill extracellular bacteria. The infected cells were then washed in PBS, lysed in 0.5% TritonX-100/PBS, and serial dilutions were plated on LB agar plates to determine the number of intracellular bacteria.

### DNA and siRNA transfection

DNA and siRNAs specific for Naip2 and Naip5 were transfected into macrophages using an Amaxa nucleofector system (Y-001 program for primary macrophages or D-032 program for cell lines) according to the manufacturers' instructions. siRNA pools for mouse Naip2 (17948; D-044151-01-04) and Naip5 (17951; D-044141-01-4) and non-targeting siRNAs were purchased from Dharmacon or synthesized by Sigma and targeting the sequences CTTACACTGAATCACAAGA (naip2) or GTGCCTTTTTAGTCCTTGT (naip5). Primer sets for RT-PCR were naip2-forward (AGGCTATGAGCATCTACCACA), naip2-reverse (AAGACATCAATCCACAGCAAA), naip5-forward (TGCCAAACCTACAAGAGCTGA), naip5-reverse (CAAGCGTTTAGACTGGGGATG), actin-forward (CATGTACGTTGCTATCCAGGC) and actin-reverse (CTCCTTAATGTCACGCACGAT). To compare caspase-1 p20 levels in immunoblotting experiments, the bands were quantified by densitometry, analyzed with ImageJ software, and normalized to the β-actin protein levels.

### Immunoprecipitation

Cell ware lysed in IP buffer [CelLytic M Cell Lysis Reagent (Sigma), 0.1 mM PMSF, and a complete protease inhibitor cocktail-EDTA (Roche) and clarified lysates were mixed with anti-T7 antibody–conjugated agarose beads (Novagen) or anti-HA conjugated sepharose beads (Covance) for 1 hr at 4°C with gentle rotation in IP buffer. Beads were washed with PBS, mixed with SDS-sample buffer and subjected to immunoblot analysis.

### Immunostaining of Asc pyroptosomes

Cells were fixed with 4% paraformaldehyde and 0.1% NP40, washed and stained with anti- Asc antibody and FITC-conjugated anti–rat antibody (Sigma) as a secondary antibody. Imaging analysis was performed using fluorescence microscopy (Olympus), and percentage of cells containing Asc pyroptosomes was determined by counting at least 300 cells in 5 separate fields.

### Asc dimerization assay

The Asc dimerization assay was previously described [25]–[27]. Briefly, cells were lysed (20 mM HEPES-KOH, pH 7.5, 150 mM KCl, 1% NP-40, 0.1 mM PMSF, and Complete protease inhibitor cocktail (Roche)) and forced onto a 21-gauge needle 10 times. The cell lysates were centrifuged at 6000 rpm for 10 min at 4°C to isolate the insoluble fraction in the pellet. The pellets were washed twice with PBS, resuspended in 500 µl of PBS and cross-linked with fresh 2 mM disuccinimidyl suberate (DTT, Sigma) for 30 min. The cross-linked pellets were isolated by centrifugation at 13000 rpm for 10 min and resuspended in 20 µl of SDS sample buffer for immunoblotting with anti-mouse Asc antibody.

### Measurements of cytokines

Mouse cytokines in culture supernatants were measured by ELISA kits (R&D Systems). Assays were performed in triplicate for each independent experiment.

### Statistical analyses

Statistical analyses were performed using the Mann–Whitney U test. Differences were considered significant at p<0.05.

## Supporting Information

Figure S1 **Naip2 promotes the processing of IL-1β in an inflammasome reconstitution system.** **A–B**. 293T cells were transfected with vector producing Nlrc4, Asc, caspase-1, pro-IL-1β, and HA-tagged Naip2 (**A**) or Naip5 (**B**). After 24 hrs, cells were infected with *Shigella* WT or S325 mutant for additional 3 hrs at a bacteria/cell ratio of 10∶1. Cell lysates were analyzed by immunoblotting with the indicated antibodies or with anti-HA for Naip2/5 detection. The level of IL-1β was further quantified by densitometry. (**A** and **B**) Results are representative of three independent experiments. *p<0.05.
(TIF)

Figure S2 **Inflammasome activation induced by** ***Shigella*** **infection is Asc- and Nlrc4- dependent, but Nlrp3-independent.** (**A**–**C**) WT, *Asc* ^−/−^, *Nlrc4* ^−/−^ or *Nlrp3* ^−/−^ BMDM were generated after differentiation for 4 days and infected with *Shigella* WT or S325 mutant at a bacteria/macrophage ratio of 10∶1 for 30 min or the indicated time points. The activation of caspase-1 was evaluated by detecting cleaved caspase-1 (p20) by immunoblotting (**A**), the production of IL-1β in cell free supernatants was analyzed by ELISA (**B**). * p<0.0001. Results represent mean ± SD. Results are representative of three independent experiments.
(TIF)

Figure S3 **WT and mutant** ***Shigella*** **uptake by BMDMs.** BMDM were infected with *Shigella* for 20 min at a bacteria/macrophage of 10∶1 and then incubated for 20 min at 37°C in medium containing gentamicin (100 µg/ml) and kanamycin (60 µg/ml) to kill extracellular bacteria. Cells were then washed in PBS, lysed in 0.5% TritonX-100/PBS and the number of intracellular bacteria evaluated by serial dilution on agar plates. The results represent mean ± SD and representative of at least three independent experiments.
(TIF)

Figure S4 **The role of Asc in pyroptosis is influenced by the time of differentiation of bone marrow-derived macrophages in culture.** BMDMs from WT and Asc^−/−^ mice were differentiated in culture for 3, 4 and 5 days, infected with *Shigella* WT or S325, and subjected to the LDH assay 2 hr post-infection. *<0.01. NS, not significant. The results represent mean and representative of at least three independent experiments.
(TIF)

Figure S5 **Pkcδ is essential for apoptosis and inhibition of apoptosis results in up-regulation of inflammasome.** (A) Activation of MAPK and NF-κB pathways in WT and *Prkcd* ^−/−^ BMDMs were assessed by western blotting using Erk1/2, MEK1/2, and IκBα phospho-antibodies at the indicated time after *Shigella* infection. (**B**–**D**) Pkcδ regulates apoptosis induced by *Shigella*. BMDMs from WT and *Prkcd* ^−/−^ mice were infected with WT *Shigella* and after 2 hrs (**B**) or 5 hrs (**C**) post-infection, cells were subjected to phosphatidylserine staining with AnnexinV (**B**) or TUNEL assay (**C**) to assess the percent of apoptotic cells in infected macrophages. * p<0.0001. Results are based on the analysis of at least 300 hundred cells in 10 microscope fields. (**D**) z-DEVD-fmk treatment enhances inflammasome activation. WT BMDMs were treated with caspase-3 inhibitor z-DEVD-fmk for 1 h, then infected with WT *Shigella*. IL-1β levels in the supernatants were measured by ELISA at 1 hr post-infection. * p = 0.0022. Results represent mean ± SD. Results are representative of at least three experiments.
(TIF)

## References

[1] Franchi L, Muñoz-Planillo R, Núñez G (2012) Sensing and reacting to microbes through the inflammasomes. Nat immunol 13: 325–332. 22430785 10.1038/ni.2231 PMC3449002

[2] Rathinam VA, Vanaja SK, Fitzgerald KA (2012) Regulation of inflammasome signaling. Nat Immunol 13: 333–2. 22430786 10.1038/ni.2237 PMC3523703

[3] Broz P, Monack DM (2011) Molecular mechanisms of inflammasome activation during microbial infections. Immunol Rev 243: 174–90. 21884176 10.1111/j.1600-065X.2011.01041.x PMC3170129

[4] Franchi L, Amer A, Body-Malapel M, Kanneganti TD, Ozören N, et al (2006) Cytosolic flagellin requires Ipaf for activation of caspase-1 and interleukin 1beta in *Salmonella*-infected macrophages. Nat Immunol 7: 576–582. 16648852 10.1038/ni1346

[5] Franchi L, Eigenbrod T, Muñoz-Planillo R, Nuñez G (2009) The inflammasome: a caspase-1-activation platform that regulates immune responses and disease pathogenesis. Nat immunol 10: 241–247. 19221555 10.1038/ni.1703 PMC2820724

[6] Lamkanfi M, Dixit VM (2012) Inflammasomes and their roles in health and disease. Annu Rev Cell Dev Biol 28: 137–61. 22974247 10.1146/annurev-cellbio-101011-155745

[7] Ashida H, Ogawa M, Kim M, Mimuro H, Sasakawa C (2011) Bacteria and host interactions in the gut epithelial barrier. Nat Chem Biol 8: 36–45. 22173358 10.1038/nchembio.741

[8] Ashida H, Ogawa M, Kim M, Suzuki S, Sanada T, et al (2011) *Shigella* deploy multiple countermeasures against host innate immune responses. Curr Opin Microbiol 14: 16–23. 20934372 10.1016/j.mib.2010.08.014

[9] Miao EA, Mao DP, Yudkovsky N, Bonneau R, Lorang CG, et al (2010) Innate immune detection of the type III secretion apparatus through the NLRC4 inflammasome. Proc Natl Acad Sci USA 107: 3076–3080. 20133635 10.1073/pnas.0913087107 PMC2840275

[10] Kofoed EM, Vance RE (2011) Innate immune recognition of bacterial ligands by NAIPs determines inflammasome specificity. Nature 477: 592–595. 21874021 10.1038/nature10394 PMC3184209

[11] Zhao Y, Yang J, Shi J, Gong Y, Lu Q, et al (2011) The NLRC4 inflammasome receptors for bacterial flagellin and type III secretion apparatus. Nature 477: 596–600. 21918512 10.1038/nature10510

[12] Halff EF, Diebolder CA, Versteeg M, Schouten A, Brondijk TH, et al (2012) Formation and structure of a NAIP5-NLRC4 inflammasome induced by direct interactions with conserved N- and C-terminal regions of flagellin. J Biol Chem 287: 38460–72. 23012363 10.1074/jbc.M112.393512 PMC3493891

[13] Qu Y, Misaghi S, Izrael-Tomasevic A, Newton K, Gilmour LL, et al (2012) Phosphorylation of NLRC4 is critical for inflammasome activation. Nature 490: 539–42. 22885697 10.1038/nature11429

[14] Schroeder GN, Hilbi H (2008) Molecular pathogenesis of *Shigella* spp.: controlling host cell signaling, invasion, and death by type III secretion. Clin Microbiol Rev 21: 134–56. 18202440 10.1128/CMR.00032-07 PMC2223840

[15] Ogawa M, Handa Y, Ashida H, Suzuki M, Sasakawa C (2008) The versatility of *Shigella* effectors. Nat Rev Microbiol 6: 11–6. 18059288 10.1038/nrmicro1814

[16] Marlovits TC, Kubori T, Sukhan A, Thomas DR, Galán JE, et al (2004) Structural insights into the assembly of the type III secretion needle complex. Science 306: 1040–1042. 15528446 10.1126/science.1102610 PMC1459965

[17] Sani M, Allaoui A, Fusetti F, Oostergetel GT, Keegstra W, et al (2007) Structural organization of the needle complex of the type III secretion apparatus of *Shigella flexneri* . Micron 38: 291–301. 16920362 10.1016/j.micron.2006.04.007

[18] Suzuki T, Franchi L, Toma C, Ashida H, Ogawa M, et al (2007) Differential regulation of caspase-1 activation, pyroptosis, and autophagy via Ipaf and ASC in *Shigella* infected macrophages. PLoS Pathog 3: 1082–1091. 10.1371/journal.ppat.0030111 PMC1941748 17696608

[19] Magdalena J, Hachani A, Chamekh M, Jouihri N, Gounon P, et al (2002) Spa32 Regulates a Switch in Substrate Specificity of the Type III Secreton of *Shigella flexneri* from Needle Components to Ipa proteins. J Bacteriol 184: 3433–3441. 12057936 10.1128/JB.184.13.3433-3441.2002 PMC135143

[20] Jouihri N, Sory MP, Page AL, Gounon P, Parsot C, et al (2003) MxiK and MxiN interact with the Spa47 ATPase and are required for transit of the needle components MxiH and MxiI, but not of Ipa proteins, through the type III secretion apparatus of *Shigella flexneri* . Mol Microbiol 49: 755–767. 12864857 10.1046/j.1365-2958.2003.03590.x

[21] Botteaux A, Kayath CA, Page AL, Jouihri N, Sani M, et al (2010) The 33 carboxyl-terminal residues of Spa40 orchestrate the multi-step assembly process of the type III secretion needle complex in *Shigella flexneri* . Microbiol 156: 2807–2817. 10.1099/mic.0.039651-0 20507885

[22] Kimbrough TG, Miller SI (2000) Contribution of *Salmonella typhimurium* type III secretion components to needle complex formation. Proc Natl Acad Sci USA 97: 11008–11013. 10984518 10.1073/pnas.200209497 PMC27139

[23] Miao EA, Rajan JV (2011) *Salmonella* and Caspase-1: A complex Interplay of Detection and Evasion. Front Microbiol 2: 85. 21833326 10.3389/fmicb.2011.00085 PMC3153046

[24] Willingham SB, Bergstralh DT, O'Connor W, Morrison AC, Taxman DJ, et al (2007) Microbial pathogen-induced necrotic cell death mediated by the inflammasome components CIAS1/Cryopyrin/NLRP3 and ASC. Cell Host Microbe 2: 147–159. 18005730 10.1016/j.chom.2007.07.009 PMC2083260

[25] Fernandes-Alnemri T, Wu J, Yu JW, Datta P, Miller B, ey al (2007) The pyroptosome: a supramolecular assembly of ASC dimers mediating inflammatory cell death via caspase-1 activation. Cell Death Differ 14: 1590–604. 17599095 10.1038/sj.cdd.4402194 PMC3345951

[26] Fernandes-Alnemri T, Alnemri ES (2008) Assembly, purification, and assay of the activity of the ASC pyroptosome. Methods Enzymol 442: 251–270. 18662574 10.1016/S0076-6879(08)01413-4

[27] Juliana C, Fernandes-Alnemri T, Wu J, Datta P, Solorzano L, et al (2010) Anti-inflammatory compounds parthenolide and bay 11-7082 are direct inhibitors of the inflammasome. J Biol Chem 285: 9792–802. 20093358 10.1074/jbc.M109.082305 PMC2843228

[28] Brown GE, Stewart MQ, Liu H, Ha VL, Yaffe MB (2003) A novel assay system implicates PtdIns(3,4)P(2), PtdIns(3)P, and PKC delta in intracellular production of reactive oxygen species by the NADPH oxidase. Mol Cell 11: 35–47. 12535519 10.1016/s1097-2765(03)00005-4

[29] Meissner F, Seger RA, Moshous D, Fischer A, Reichenbach J, et al (2010) Inflammasome activation in NADPH oxidase defective mononuclear phagocytes from patients with chronic granulomatous disease. Blood 116: 1570–1573. 20495074 10.1182/blood-2010-01-264218 PMC2938844

[30] Basu A (2003) Involvement of protein kinase C-delta in DNA damage-induced apoptosis. J Cell Mol Med 7: 341–50. 14754503 10.1111/j.1582-4934.2003.tb00237.x PMC6740315

[31] Brodie C, Blumberg PM (2003) Regulation of cell apoptosis by protein kinase c delta. Apoptosis 8: 19–27. 12510148 10.1023/a:1021640817208

[32] Reyland ME, Anderson SM, Matassa AA, Barzen KA, Quissell DO (1999) Protein kinase C delta is essential for etoposide-induced apoptosis in salivary gland acinar cells. J Biol Chem 274: 19115–23. 10383415 10.1074/jbc.274.27.19115

[33] Mariathasan S, Hewton K, Monack DM, Vucic D, French DM, et al (2004) Differential activation of the inflammasome by caspase-1 adaptors ASC and Ipaf. Nature 430: 213–218. 15190255 10.1038/nature02664

[34] Kanneganti TD, Body-Malapel M, Amer A, Park JH, Whitfield J, et al (2006) Critical role for cryopyrin/Nalp3 in activation of caspase-1 in response to viral infection and double-stranded RNA. J Biol Chem 281: 36560–36568. 17008311 10.1074/jbc.M607594200

[35] Ozoren N, Masumoto J, Franchi L, Kanneganti TD, Body-Malapel M, et al (2006) Distinct roles of TLR2 and the adaptor ASC in IL-1beta/IL-18 secretion in response to *Listeria monocytogenes* . J Immunol 176: 4337–4342. 16547271 10.4049/jimmunol.176.7.4337

[36] Sasakawa C, Kamata K, Sakai T, Murayama Y, Makino S, et al (1986) Molecular alteration of the 140-megadalton plasmid associated with loss of virulence and congo red binding activity in *Shigella flexneri* . Infect Immun 51: 470–475. 3002985 10.1128/iai.51.2.470-475.1986 PMC262355

[37] Watarai M, Tobe T, Yoshikawa M, Sasakawa C (1995) Contact of *Shigella* with host cells triggers release of Ipa invasins and is an essential function of invasiveness. EMBO J 14: 2461–2470. 7781600 10.1002/j.1460-2075.1995.tb07243.x PMC398359

[38] Kodama C, Matsui H (2004) *Salmonella* flagellin is not a dominant protective antigen in oral immunization with attenuated live vaccine strains. Infect Immun 72: 2449–2451. 15039380 10.1128/IAI.72.4.2449-2451.2004 PMC375176

[39] Franchi L, Kamada N, Nakamura Y, Burberry A, Kuffa P, et al (2012) NLRC4-driven production of IL-1β discriminates between pathogenic and commensal bacteria and promotes host intestinal defense. Nat Immunol 13: 449–456. 22484733 10.1038/ni.2263 PMC3361590

[40] Suzuki Y, Yoshitomo-Nakagawa K, Maruyama K, Suyama A, Sugano S (1997) Construction and characterization of a full length-enriched and a 5′-end-enriched cDNA library. Gene 200: 149–156. 9373149 10.1016/s0378-1119(97)00411-3

[41] Adami C, Brunda MJ, Palleroni AV (1993) In vivo immortalization of murine peritoneal macrophages: a new rapid and efficient method for obtaining macrophage cell lines. J Leukoc Biol 53: 475–8. 7683328 10.1002/jlb.53.4.475

